# Integrating Natural Language Processing in Child and Adolescent Mental Health Services Research Using Normalization Process Theory: Implementation Report

**DOI:** 10.2196/96370

**Published:** 2026-10-26

**Authors:** Huilin Chen, Jennifer Chapman, En-Nien Tu, Siyu Zhou, Jasmine Laing, Emma Fergusson, Andrey Kormilitzin

**Affiliations:** 1 Department of Psychiatry University of Oxford Oxford, England United Kingdom; 2 Oxford Health NHS Foundation Trust Oxford, England United Kingdom; 3 Department of Experimental Psychology University of Oxford Oxford, England United Kingdom; 4 Bay Area School of Applied Psychology Sciences Beijing Normal University Zhuhai China

**Keywords:** artificial intelligence, AI, electronic health records, child and adolescent mental health services, natural language processing, local language models

## Abstract

**Background:**

Anxiety problems are prevalent among autistic children and adolescents and those with attention-deficit/hyperactivity disorder (ADHD). Natural language processing (NLP) is increasingly used to structure information from electronic health records (EHRs), but its implementation within secure child and adolescent mental health services (CAMHS) research environments is less frequently reported.

**Objective:**

This study documented the specification, governance, integration, and evaluation of an NLP-enabled information extraction workflow within the Oxford Health National Health Service (NHS) Foundation Trust CAMHS research setting for extracting documented anxiety treatment information and outcome-related text from the EHRs of autistic young people and those with ADHD and examined implementation using normalization process theory (NPT).

**Methods:**

A multidisciplinary team developed a locally deployed NLP pipeline to identify labeled text spans describing presenting problems, medication attributes, psychological therapies, and treatment outcomes in pseudonymized EHR notes. Implementation followed an iterative seven-phase road map: (1) defining research questions, (2) identifying key constructs, (3) codeveloping an annotation schema, (4) training human annotators, (5) resolving annotation discrepancies, (6) large-scale annotation of EHR data, and (7) developing and evaluating the NLP model. NPT was applied throughout to guide implementation and evaluate progress. Quantitative outcomes (data volume processed, annotation reliability, and model performance) and qualitative feedback were collected to assess implementation success.

**Implementation (Results):**

The source cohort comprised 454,127 notes from 5906 patients; 697 (0.2%) randomly selected, manually annotated notes from 302 (5.1%) patients were used for model development and held-out evaluation. Category-specific position-based Krippendorff α values ranged from 0.325 to 0.579 for problem, psychological therapy, and outcome spans and from 0.665 to 0.967 for medication spans. Agreement was highest for medication names, doses, and frequencies and lowest for cognitive problems and treatment outcomes. Held-out macroaveraged F1-score was 0.87 (95% CI 0.86-0.89) across the 4 medication labels and 0.71 (95% CI 0.68-0.73) across the 5 problem labels, psychological therapy labels, and outcome labels; label-specific F1-score ranged from 0.60 to 0.98. Team-generated implementation records indicated that multidisciplinary schema development and clear protocols supported shared understanding and engagement, whereas annotation burden, documentation variability, data access delays, and computing constraints impeded implementation. No major adverse events or harms were reported.

**Conclusions:**

This report demonstrates the feasibility of integrating NLP tools into NHS CAMHS research workflows to extract structured information on anxiety treatments from routine clinical records. The NPT framework provided a structured approach for documenting the organizational work, barriers, and adaptations involved. External validation, refinement of less reliable labels, and further evaluation are required before broader use.

**International Registered Report Identifier (IRRID):**

RR2-10.31234/osf.io/r4gyw

## Introduction

### Context

Anxiety problems are common among autistic children and young people and those with attention-deficit/hyperactivity disorder (ADHD), and their prevalence increases with the complexity of co-occurring autism and ADHD, representing a substantial public health concern [1].

Despite clear clinical needs, research evidence on effective treatments for anxiety problems in neurodivergent children and adolescents remains inconclusive. A network meta-analysis found that cognitive behavioral therapy (CBT) was associated with lower posttreatment anxiety severity in autistic children, although a similar effect was not evident when examining changes in scores from baseline [2]. In children with ADHD, evidence is limited and mixed regarding the impact of first-line stimulant medications on anxiety outcomes. Some small-scale studies suggest benefits, while others indicate potential exacerbation of anxiety [3]. Similarly, other interventions (eg, the nonstimulant medication atomoxetine, selective serotonin reuptake inhibitors, and psychosocial therapies, including CBT) have a promising but very limited evidence base, with findings of treatment benefits in these groups still mixed [3-5]. These gaps call for practice-based investigation of how anxiety is documented and managed in routine care for autistic young people and those with ADHD.

### Problem Statement

Within the UK National Health Service (NHS) child and adolescent mental health services (CAMHS) setting, long wait times for neurodevelopmental assessments and mental health care indicate substantial pressure on services and leave service evaluation and implementation research deprioritized. Clinically relevant information is often recorded in free text rather than consistently represented in structured fields, making manual review impractical at scale. These factors are major barriers to translating research into practice [6]. This project therefore used natural language processing (NLP) to convert selected information in CAMHS electronic health records (EHRs) into structured variables for research on documented anxiety treatment and outcomes in neurodivergent children and young people.

### Similar Interventions

Traditionally, evidence generation in CAMHS has relied on prospective research (randomized trials and observational studies) and retrospective service evaluations, which can be slow and resource-intensive. Controlled studies may not represent routine practice or heterogeneous clinical populations, while service evaluations typically measure end points (eg, service performance) rather than the practices and contextual factors that influence long-term success in real-world settings.

Approaches using routinely collected clinical data are now established as complements to prospective studies and manual service evaluations. Automated text mining and NLP have been used to extract clinical information from EHRs for research purposes in other domains of psychiatry [7-9]. However, little is known about how such tools are implemented, governed, and integrated within mental health services research settings. Normalization process theory (NPT) [10,11] conceptualizes implementation through coherence, cognitive participation, collective action, and reflexive monitoring and has been applied within an NHS context [12]. This implementation report therefore aims to document how an NLP-enabled information extraction (IE) workflow was specified, governed, embedded, and evaluated within NHS CAMHS research and to identify facilitators and barriers to its normalization using NPT.

## Methods

### Research Questions Informing the Implementation

The NLP workflow was developed to support an underlying clinical research program addressing the following questions:

- Do autistic children and young people, and/or children and young people with ADHD receive treatment for documented anxiety problems, and, if so, what treatments are recorded?
- What responses to these treatments are documented in routine clinical notes?
- What label-specific precision, recall, and F_1_-scores does a locally deployed span-categorization model achieve when extracting the prespecified clinical constructs from EHR?
- How do documented outcomes vary across different treatment modalities (psychological, pharmacological, or combined)?

The results of this analysis will be reported separately. The original plan was to capture presenting problems strictly according to diagnostic criteria. This was revised because relevant information was frequently documented in formulation-led free text rather than as coded diagnoses or complete symptom lists.

### Blueprint Summary

We implemented the NLP workflow through a 7-phase iterative road map (Figure 1) that combined technical development with integration into clinical research practices. First, the team defined the research questions. As a multidisciplinary team, clinicians and researchers identified questions about documented anxiety treatments and outcomes that could be addressed using available EHR data. As a result of this phase, the initial focus on formally coded anxiety diagnoses was broadened to include relevant manifestations documented in free text.

**Figure 1:**
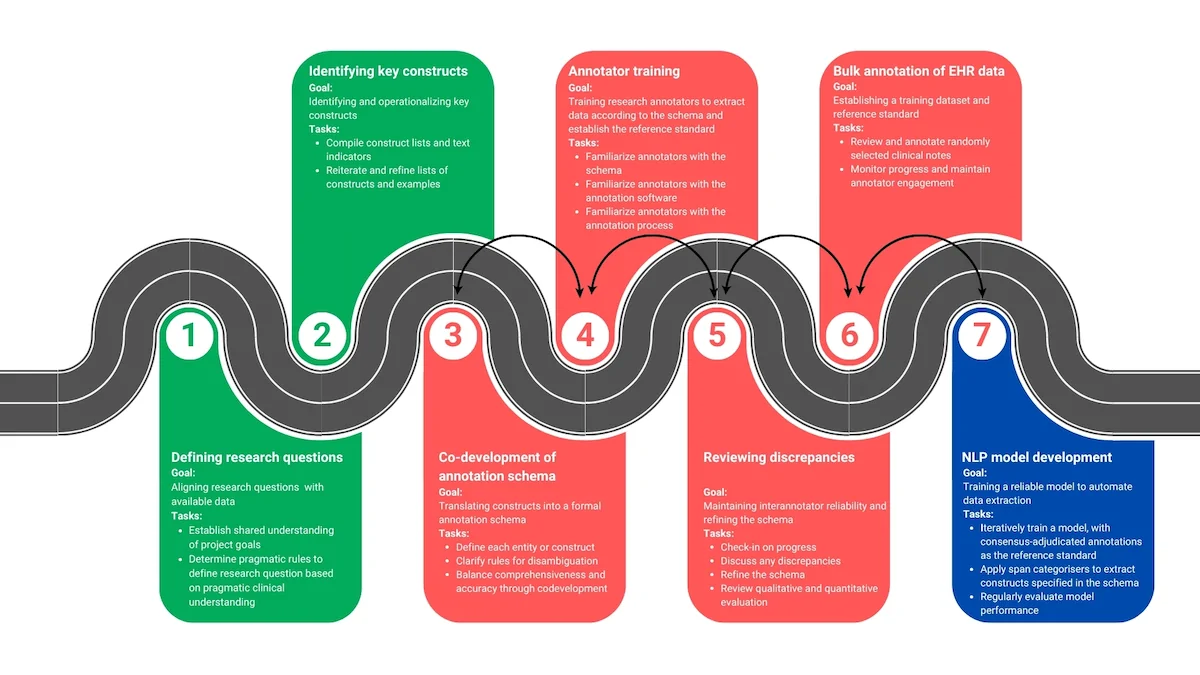
Implementation road map illustrating the 7 key phases of the project.

Second, the team identified key constructs. The multidisciplinary team operationalized the definitions of presenting problems, types of anxiety interventions, and clinical response constructs such as symptom improvement, with side effects represented by a separate annotation category. Team members collaboratively and iteratively refined the list of key constructs and examples, thereby increasing stakeholder investment (“cognitive participation”).

Third, the team codeveloped the annotation schema. The constructs were translated into span labels with definitions, boundary rules, and disambiguation guidance. Pilot annotation identified additional concepts, including nonpharmacological support (eg, “buddy programs”). The final annotation schema, decision rules, and synthetic worked examples are provided in Multimedia Appendix 1; the schema was refined over 6 consensus rounds.

Fourth, the team trained the annotators. Four doctoral-level clinical researchers were trained to identify and label text spans according to the schema using an annotation platform. Training included written guidance, worked examples, and practical workshops, catering to nontechnical team members who faced a learning curve in using the annotation platform and understanding machine learning-oriented thinking (“cognitive participation challenge”).

Fifth, the team reviewed discrepancies. Preadjudication agreement was monitored using Krippendorff α. Annotators discussed disagreements in regular consensus meetings and workshops, after which definitions, boundary rules, and examples were clarified where required, providing opportunities for “reflexive monitoring.”

Sixth, the team annotated the EHR data. Over 4 calendar months, 4 clinical researchers reviewed and annotated 970 clinical notes through the Clinical Record Interactive Search (CRIS) platform. Each note was annotated by at least 2 researchers.

Finally, the team developed and iteratively trained the model. Consensus-adjudicated annotations were used as the reference standard for training a span categorizer machine learning model to identify and label clinical constructs. The final model was evaluated against a held-out test set of annotated notes using strict label-and-boundary matching, precision, recall, and *F*_1_-scores.

The NPT framework informed implementation throughout the 7 phases. The team regularly discussed how our understanding (“coherence”) evolved, how engagement (“cognitive participation”) was fostered or impeded, what actions and operational work (“collective action”) were required, and how we appraised progress (“reflexive monitoring”). We also documented any adaptations made to the original plan as part of normalizing the new processes into our workflow.

### Technical Architecture and Key Features of the NLP Tool

#### Processing Environment

The pipeline processed pseudonymized free-text clinical notes and returned structured records containing the predicted label, character-level start and end offsets, source note identifier, and associated metadata. All processing was performed within a trusted research environment.

#### Data Platform

Clinical notes were accessed through the CRIS system on the Akrivia Health platform [13]. The source data and NLP pipeline were hosted within the same secure Amazon Web Services (AWS; Amazon Inc) virtual desktop environment. Patient-level free text was not transferred outside the TRE.

#### Software

The pipeline was implemented in Python (version 3.10; Python Software Foundation; Multimedia Appendix 2 provides details on all software), using spaCy’s (Explosion AI) SpanCategorizer for supervised span identification and labeling [14], PyTorch (Meta Platforms Inc) [15] and Hugging Face Transformers for model training [16], and *scikit-learn* and custom Python code for evaluation. The evaluated medication, problem, psychological therapy, and outcome targets were all represented as span-categorization tasks; no document-level multilabel classifier was used. This in-house approach allowed the annotation schema and model configuration to be adapted to the study’s clinical constructs and secure computing environment. Previous work by the group informed the development approach [17-22].

### Availability and Intended Use

The annotation schema is provided in Multimedia Appendix 1. Code and model configuration files are available to researchers with the required CRIS approvals within the secure environment. The pipeline was developed for secondary research and has not been validated for clinical decision-making. The intellectual property of the pipeline is owned by the University of Oxford and managed operationally by the Oxford Health NHS Foundation Trust (OHFT) CRIS team. Deployment in another organization or for clinical use would require external validation, assessment of generalizability, and local information governance and regulatory review.

### Target Population and Setting

#### Implementation Target

The primary target of this NLP tool was the research process within an NHS CAMHS context. Specifically, researchers and staff aimed to improve the efficiency and scope of data analysis for a study on anxiety problems in neurodivergent youth. The intended beneficiaries of the knowledge gained are service users and clinicians working with children and young people with autism and/or ADHD and anxiety problems, by supporting subsequent governed observational analyses. The immediate users of the NLP tool are clinical researchers in our team who have access to CAMHS EHR data, as the tool was designed to enhance their capabilities in extracting and analyzing information.

#### Implementation Setting

This project was implemented at the OHFT, a public mental health care provider in England. OHFT’s services cover several counties (Oxfordshire, Buckinghamshire, Swindon, Wiltshire, Bath, and North-East Somerset), including community CAMHS clinics.

#### Impacts of the Implementation

The full annotation schema covered 4 broad information domains: presenting problems, treatment provision, outcome-related information, and working diagnoses [23]. The model evaluation reported here was restricted to 11 span labels covering medication attributes, problems, psychological therapy, and outcome-related information. Working diagnosis and 5 other schema labels were not included in the evaluated model. The evaluated domains were selected because they addressed the prespecified research questions and were not consistently available in structured EHR fields. Detailed analyses of documented treatment provision and outcome-related information will be reported separately.

### Research Target

The intended study population comprised children and young people (aged 6-18 years) with evidence of autism and/or ADHD and evidence of anxiety difficulties [23]. To maximize sensitivity during initial record retrieval, the OHFT CRIS team first created a broad candidate cohort using a union criterion that contained evidence of autism or ADHD, evidence of anxiety, or both (Multimedia Appendix 2).

The present implementation study evaluates the development of the extraction workflow within the broad candidate cohort; analyses of clinical findings in the final both-criteria clinical cohort will be reported separately. The OHFT CRIS data team applied these criteria using SQL to identify the eligible cohort and retrieve associated pseudonymized clinical notes. The broad candidate cohort contained 454,127 notes from 5906 patients who received CAMHS care within the OHFT between 2011 and 2022. From this source cohort, 970 unique notes from 426 patients were selected for annotation using random sampling at the patient level. Ten notes from 7 patients were used for schema piloting and excluded from quantitative analyses. Of the remaining 960 notes, 263 notes from 117 patients were allocated to the interannotator reliability corpus and were independently reviewed by 4 annotators; the remaining 697 notes from 302 patients formed the model corpus.

### Data Governance and Privacy

All data used (clinical notes and structured fields) were owned by the OHFT and accessed through the AWS environment used by Akrivia Health, a platform designed for secondary use of pseudonymized patient data. Researchers could only access EHRs via a secure virtual desktop infrastructure (VDI) on AWS, which prevented any patient identifiable information from being removed or exposed externally. Within this environment, all data processing (ie, manual annotation and automated NLP) was performed.

Data life cycle considerations were addressed as follows: clinical data were collected in routine care and then processed for research by Akrivia Health following an automatic pseudonymization step. The pseudonymized data were stored in the secure AWS workspace. No data were downloaded to personal computers. Researchers only accessed the pseudonymized dataset without modification to the source data. The OHFT CRIS team oversees all research projects and regularly audits data use on the Akrivia Health platform.

### Interoperability With Clinical Systems

The Akrivia Health platform we used aggregates data from 2 EHR systems used in the Trust (ie, CareNotes [OneAdvanced] and RiO [The Access Group]). Akrivia Health itself handles the extraction and pseudonymization of data from those systems and provides data in a standardized format (both structured fields and text). Our text mining NLP IE pipeline was developed to process this CRIS-formatted data. In practical terms, this meant our data input scripts were tailored to the database schema and data tables of CRIS (eg, knowing how encounters, patients, and notes are linked via IDs).

### NLP Model Development and Evaluation

#### Target Concepts

All reported targets were annotated as labeled text spans with character-level start and end offsets and were formulated as span-categorization tasks. Medication labels comprised drug name, dose, action, and frequency. Problem labels comprised cognitive, emotional, social, biological, and other problems, with other treated as 1 aggregated problem category. Additional labels identified psychological therapy and treatment outcomes. Table 1 summarizes the modeled labels and their frequencies. Additional categories retained in the annotation schema for descriptive annotation or future model development are listed in Table 1 and Multimedia Appendix 1. Technical details of model architecture and data handling are provided in Multimedia Appendix 2.

**Table 1:** Distribution of annotated examples across model development and test sets used to develop the natural language processing model and evaluate its performance^a^.

Categories			Model development, n		Test set, n
Patients			226		76
Notes			549		148
**Medication treatment**					
	Drug names	1774		749	
	Dose	894		372	
	Action	632		259	
	Frequency	791		333	
**Problems**					
	Cognitive	146		43	
	Emotional	360		141	
	Social	117		37	
	Biological	92		34	
	Other	591		221	
Psychological therapy			158		53
Outcomes			182		79

^a^All counts for clinical labels represent annotated reference spans.

#### Model Architecture and Evaluation Protocol

All 11 targets were implemented using spaCy’s SpanCategorizer [14], with a ROBERTa (Meta AI)-based encoder [24], the standard pretrained weights of which were obtained through Hugging Face Transformers [16,24]. The SpanCategorizer and encoder were fine-tuned jointly on the consensus-adjudicated annotations in the model development partition; long notes were processed into paragraph-based chunks, and predicted offsets were mapped back to the source note. The 697-note model corpus was split at the patient level into 549 model development notes (from 226 patients) and 148 held-out test notes (from 76 patients), with no patient in both partitions; internal model selection used a note-level 440/109 split of the development notes and a fixed decision threshold of 0.5.

Interannotator reliability was estimated using position-based nominal Krippendorff α on the 263 notes reviewed by all 4 annotators. The held-out test set was used once to estimate strict span-level precision, recall, and *F*_1_-score (with label and both character boundaries exactly matching a reference span), with 95% CIs from 5000 patient-level cluster bootstrap replicates. Full details are provided in Multimedia Appendices 2 and 3.

Regular NPT-informed structured team reflection and evaluation were facilitated based on: (1) an implementation journal documenting key decisions, changes, and rationales, coded prospectively according to the 4 NPT constructs; (2) minutes from regular team and annotator consensus meetings; and (3) quantitative process indicators, including annotation agreement, model performance, and timeline deviations. These records were reviewed deductively against the 4 NPT constructs of coherence, cognitive participation, collective action, and reflexive monitoring. Initial categorization was undertaken by HC and subsequently discussed with the wider research team. Differences in interpretation were discussed during team meetings. Key themes were recorded by HC after each team meeting in the implementation journal. Main findings were summarized in the following Implementation Process Outcomes subsection.

### Ethical Considerations

This study used pseudonymized routinely collected EHR data from the OHFT CRIS system. The Oxford Health CRIS research database has approval from the South Central-Oxford C Research Ethics Committee (15/SC/0247). The present study was reviewed and approved by the Oxford Health CRIS Oversight Group. As the study involved secondary analysis of pseudonymized data within the approved CRIS research database framework, a separate project-specific NHS Research Ethics Committee application was not required. All analyses were conducted within the approved TRE. The examples in Multimedia Appendix 1 are synthetic composites; no verbatim clinical record text is reproduced.

## Implementation (Results)

### Outcomes of the Implementation

#### Annotated Corpus and Interannotator Agreement

The 960 notes from 419 patients comprised 697 notes from 302 patients used for model development and evaluation, and 263 notes from 117 patients assigned to interannotator reliability analysis. Category-specific reliability differed substantially across constructs. For nonmedication labels, Krippendorff α was 0.579 (95% CI 0.477-0.635) for emotional problems, 0.546 (95% CI 0.409-0.699) for psychological therapy, 0.516 (95% CI 0.280-0.688) for social problems, 0.418 (95% CI 0.271-0.467) for other problems, 0.411 (95% CI 0.318-0.526) for biological problems, 0.343 (95% CI −0.028 to 0.549) for outcomes, and 0.325 (95% CI 0.096-0.418) for cognitive problems. Medication label reliability was higher: 0.967 (95% CI 0.955-0.979) for drug name, 0.927 (95% CI 0.881-0.965) for frequency, 0.926 (95% CI 0.892-0.959) for dose, and 0.665 (95% CI 0.579-0.743) for medication action. All estimates are reported in Multimedia Appendix 3.

The model development and evaluation corpus contained 494,963 words; the median note length was 527 (IQR 261-925) words. Before model training, the 697-note model corpus was divided at the patient level into a 549-note model development partition and a 148-note held-out test partition. The complete model architecture and training configuration are specified in Multimedia Appendix 2.

#### Technical Evaluation Outcomes

Using strict label-and-boundary matching on the held-out test set, medication span *F*_1_-score was 0.98 for drug names, 0.92 for doses, 0.91 for frequencies, and 0.66 for medication action (Table 2). For problem, psychological therapy, and outcome spans, label-specific *F*_1_-score ranged from 0.60 to 0.86 (Table 3). Performance was highest for drug names and the aggregated other-problem label and lowest for cognitive and social problems, biological problems, medication action, and outcomes. These results demonstrate substantial variation across labels, which cannot be represented by a single overall *F*_1_-score.

Strict span-categorization performance varied across the broader clinical constructs. Emotional problems and the aggregated other-problem label had the highest *F*_1_-scores, whereas cognitive, social, and biological problems and treatment outcomes had lower performance. The wider CIs for several labels reflect greater uncertainty in the less frequent categories (Table 3).

In terms of efficiency, the model processed approximately 3 notes per second (Intel Xeon Platinum 8488C, 2.4 GHz, 32 GB RAM; Intel Corp), vs approximately 4 minutes per note for a human annotator, representing an approximately 700-fold increase in throughput, supporting practical viability for large-scale observational research. This operational comparison indicates substantially higher processing throughput than manual annotation under the reported environment.

**Table 2:** Strict span-categorization performance for medication names and medication attribute spans^a^.

Labels	Precision (95% CI)	Recall (95% CI)	*F*_1_-score (95% CI)
Drug name^b^	0.97 (0.97-0.98)	0.98 (0.97-0.99)	0.98 (0.97-0.98)
Dose^c^	0.92 (0.91-0.93)	0.91 (0.91-0.93)	0.92 (0.91-0.93)
Action^d^	0.66 (0.64-0.69)	0.65 (0.63-0.68)	0.66 (0.64-0.68)
Frequency^e^	0.89 (0.88-0.91)	0.93 (0.92-0.95)	0.91 (0.91-0.93)
Macroaverage^f^	0.86 (0.84-0.88)	0.87 (0.84-0.88)	0.87 (0.86-0.89)

^a^Values are observed held-out test set estimates with 95% percentile CIs obtained from 5000 patient-level cluster bootstrap replicates. A prediction was considered correct only when its label and start and end character offsets exactly matched those of the reference span.

^b^Drug name denotes the medication mentioned (eg, sertraline).

^c^Dose denotes the amount per administration (eg, “5 mg”).

^d^Action denotes a prescribing action (eg, initiation, continuation, withholding, or discontinuation).

^e^Frequency denotes how often the medication was prescribed (eg, “twice daily”).

^f^Macroaverages are the unweighted means of the 4 label-specific metrics.

**Table 3:** Strict span-categorization performance for problems, psychological therapy, and treatment outcomes on the held-out test set^a^.

Labels			Precision (95% CI)		Recall (95% CI)		*F*_1_-score (95% CI)
**Problems**							
	Cognitive	0.57 (0.48-0.67)		0.64 (0.54-0.72)		0.6 (0.52-0.67)	
	Emotional	0.78 (0.72-0.83)		0.91 (0.87-0.95)		0.84 (0.80-0.88)	
	Social	0.58 (0.48-0.69)		0.63 (0.52-0.73)		0.60 (0.51-0.68)	
	Biological	0.74 (0.62-0.86)		0.52 (0.39-0.63)		0.61 (0.50-0.70)	
	Other	0.81 (0.76-0.86)		0.91 (0.87-0.95)		0.86 (0.82-0.89)	
Psychological therapy			0.68 (0.60-0.76)		0.85 (0.78-0.92)		0.76 (0.69-0.81)
Outcomes			0.66 (0.57-0.75)		0.68 (0.60-0.77)		0.67 (0.60-0.74)
Macroaverage^b^			0.69 (0.67-0.73)		0.73 (0.71-0.77)		0.71 (0.68-0.73)

^a^Values are observed held-out test set estimates with 95% percentile CIs obtained from 5000 patient-level cluster bootstrap replicates. A prediction was correct only when its label and start and end character offsets exactly matched those of the reference span.

^b^Macroaverages are the unweighted means of the 7 label-specific metrics.

#### Implementation Process Outcomes

Using NPT as a guiding framework, we summarized observations about how NLP was understood, enacted, appraised, and integrated within our team’s workflow (Table 4). No major adverse effects or unintended harms were reported.

**Table 4:** Normalization process theory domains and their relevance to the implementation.

Domains	Coherence	Cognitive participation	Collective action	Reflexive monitoring
Definitions	How team members perceive and comprehend the purpose, value, and implications of NLP^a^ approaches to analysis of electronic health records in CAMHS^b^	The relational work required to build and sustain engagement with NLP approaches	The operational work required to implement NLP approaches within the CAMHS research workflow effectively	The appraisal work that individuals and teams do to assess the impact of NLP approaches
Key outcomes	By the end of the project, implementation journal records and meeting minutes both indicated that the team had a strong shared understanding of the purpose and value of the NLP tool. This was mainly achieved through the early collaborative engagement in defining research questions and the schema, which allowed everyone to understand the rationale of the research project and our current design.	Engagement was generally high. The multidisciplinary nature of the team was a strength, as it sparked discussions and cross-learning from different perspectives. Team members, particularly clinicians, were more engaged with data-driven methods, which was an intended culture shift.	This domain saw both smooth coordination and operational hurdles. One opportunity the NLP approach provided was the structured road map, which provided clear definitions of individual roles and responsibilities (annotation, coding, and evaluation) across the project’s life cycle.	We scheduled regular reflective team meetings, which provided room and opportunities for the team to reflect on what had or had not worked and how to move forward. We also quantitatively monitored some aspects (annotation agreement and model performance) and shared those results. These regular discussions enabled efficient reflexive monitoring throughout the project.
Key challenges	A challenge to coherence was the abstract nature of NLP for those unfamiliar with either the technical principles or the software setup. Due to the sophisticated nature of our target constructs, we also experienced setbacks on improving model performance beyond a certain threshold to extract and categorize heterogeneous information with sufficient sensitivity and specificity.	We experienced some reduction in engagement during the lengthy annotation phase.	A significant challenge in collective action was related to infrastructural constraints, where significant unexpected time lags occurring during the data access process and inadequate computing power leading to disruptions to model training. This external dependency affected the team’s overall momentum. The time and labor intensity of NLP development was another main challenge.	Although reflexive monitoring was instrumental to the success of our project, we also pivoted multiple times due to trial and error. However, every pivot in our modeling or conceptualizing approaches (eg, whether we chose to use named-entity recognition or a local language model with prompt engineering) meant that previously completed work had to be necessarily redone, leading to high costs of reconfiguration.

^a^NLP: natural language processing.

^b^CAMHS: child and adolescent mental health services.

### Lessons Learned

Throughout this project, we identified the following strategies to address our main challenges (Table 5).

**Table 5:** Key strategies.

Key strategies	Description
Flexibility and iteration	We learned that flexibility in approach is crucial. For example, we had to rescope our research question from coded diagnoses to free-text constructs. Rather than seeing such pivots as setbacks, treating them as part of an agile implementation process helped us manage expectations. With the NLP^a^ approach, being prepared to pivot is essential, whether when trying a new algorithm or redefining a construct. Hence, an iterative, learning-oriented implementation strategy (with NPT’s^b^ reflexive monitoring) proved valuable.
Interdisciplinary collaboration	A major success factor was the close collaboration between clinicians and researchers. Early and ongoing interdisciplinary dialogue (especially during schema development) helped bridge the gap between clinical context and technical execution. This ensured the tool stayed relevant to real-world needs and that the team built a sense of joint ownership. Future projects should invest time in this co-design process; in our project, such a co-design process surfaced constructs such as the “buddy program” that the initial schema had missed.
Ensuring adequate technical infrastructure	One of the challenges that significantly impacted our timeline was securing appropriate IT infrastructure. We initially underestimated the complexity of working within secure hospital systems: everything from gaining server permissions to ensuring adequate computing power took longer than anticipated. The lesson is to involve IT stakeholders early and plan for potential delays or needs (eg, arranging backup compute options). Also, ensuring the project has a designated priority or sponsor within the IT department can help accelerate necessary support.
Rigorous data preparation and pragmatic understanding of data	We discovered that a lot of effort was spent on understanding and cleaning the data (even in pseudonymized form). Inconsistent documentation practices by clinicians meant our NLP had to handle a wide variety of expressions. Some outcomes we sought were not consistently documented at all. Much of the project’s effort went into understanding and mitigating inconsistencies in outcome documentation across the dataset. For future projects, conducting a thorough initial data assessment (and if possible, engaging clinicians to improve documentation practices in parallel) could improve outcomes. In our case, we captured this as a recommendation: encouraging clinicians to document key outcome information in structured ways could complement NLP efforts.
Resource and time realism	We learned to be realistic about the time commitment required. The annotation process took over 4 calendar months; fortunately, we had a team to distribute this, but it still led to some fatigue. If someone were implementing a similar project without a team of annotators, it could stall. So, either securing sufficient manpower or exploring techniques such as active learning (where the model helps reduce annotation load) would be wise. This project leveraged the deidentified Clinical Record Interactive Search dataset and Akrivia’s platform, both of which were free for approved research use. We note that we were fortunate to mostly stay within budget for human resources but only because of careful scope management (we did not try to do too many things at once). The lesson is to align the project scope with available resources and perhaps build in a cushion for unexpected hurdles.
Thorough documentation	The practice of good documentation (both of technical steps and decision rationales) was an important facilitator when team members rotated or when we wrote up results. We maintained an implementation journal logging key decisions, changes, and reasoning aligned to NPT domains. This habit is something we would recommend to any implementation team—it makes writing the final report easier and supports knowledge transfer if others adopt the approach.

^a^NLP: natural language processing.

^b^NPT: normalization process theory.

## Discussion

This implementation report shows that an NLP-enabled workflow can be embedded within a secure NHS CAMHS research environment to convert selected information in free-text notes into structured research variables. Multidisciplinary schema development, consensus work, secure infrastructure, and ongoing appraisal supported implementation, whereas annotation burden, documentation variability, data access delays, and computing constraints required adaptation. Technical performance, treated as an implementation feasibility outcome, varied by construct: medication names, doses, and frequencies were annotated and extracted more consistently than medication actions, broader problem categories, and treatment outcomes. The resulting dataset is intended to support observational research; the pipeline has not yet been validated for clinical decision-making.

The evaluated pipeline represents a preparatory IE component rather than a complete treatment response analysis. Because medication, attribute, therapy, and outcome spans were extracted independently, answering the downstream clinical questions will require additional note- or patient-level aggregation, resolution of treatment attribute and treatment outcome relationships, and validation of the resulting derived variables.

The road map and NPT-informed documentation provided transferable lessons for integrating advanced analytics within research practice. Mapping activities and observations to coherence, cognitive participation, collective action, and reflexive monitoring showed where implementation progressed, encountered barriers, and required adaptation. Repeated pivots also illustrated the value of an iterative, learning-oriented approach, rather than being considered as implementation failures.

Several limitations should be considered. First, this was a single implementation within 1 NHS Trust, so the transferability of findings may be limited by differences in populations, documentation practices, infrastructure, and governance. We followed the iCHECK-DH (Guidelines and Checklist for the Reporting on Digital Health Implementations) reporting guidance [25] to support transparency (Multimedia Appendix 4). Second, the NPT-informed evaluation relied partly on team-generated records and interpretations, and stakeholder acceptability and feasibility were not measured using validated instruments. Third, documentation and annotation ambiguity varied by construct. Medication names, doses, and frequencies generally had clearer lexical boundaries than problem categories and treatment outcomes. Category-specific Krippendorff α ranged from 0.325 to 0.579 for nonmedication constructs and from 0.665 to 0.967 for medication attributes; for outcomes, Krippendorff α was 0.343 (95% CI −0.028 to 0.549), indicating substantial uncertainty and not excluding chance-level agreement. Lower reliability may reflect both ambiguity in the clinical constructs and their documentation, as well as inconsistency in applying the operational definitions. Model performance measures agreement with the consensus-adjudicated reference annotations and should therefore be interpreted in light of these sources of ambiguity; it does not directly quantify construct ambiguity or establish clinical validity. Fourth, the 549-note model development partition was split at the note level, so notes from the same patient could occur in both the training and internal development subsets, although the held-out test set was patient disjoint. Fifth, the model was trained once using a single random seed. The patient-level bootstrap CIs reflect patient resampling in the test set, not training variability. Finally, the model evaluation remained local and research-only, without external or clinical validation for nonresearch purposes (eg, as a clinical decision tool). The model extracted spans independently and did not link medication attributes or outcomes to treatments or classify outcome polarity or temporality. No dictionary-based, rule-based, or alternative machine learning baseline was evaluated; the results therefore establish local feasibility and label-specific performance, but they do not imply superiority over simpler extraction methods or alternative model architectures.

In conclusion, an NLP-enabled workflow can structure selected mental health information from clinical records at scale, but implementation requires multidisciplinary co-design, iterative definitions, realistic resourcing, secure infrastructure, and ongoing appraisal. Refinement of the definitions and extraction methods for less reliable labels, and evaluation in other settings are required before broader use.

## Data Availability

The data used in this study were obtained from the Oxford Health National Health Service (NHS) Foundation Trust Clinical Record Interactive Search (CRIS) system and consist of pseudonymized electronic patient records. Researchers may request access through the CRIS administrative team, subject to approval. Further information is available at the Oxford Health NHS foundation trust website [27].

## Appendix

Multimedia Appendix 1 Supporting autistic children and young people with anxiety problems (ACECAT)-Clinical Record Interactive Search (CRIS) annotation schema and worked example.

Multimedia Appendix 2 Technical specification of the natural language processing pipeline and evaluation.

Multimedia Appendix 3 Category-specific Krippendorff α for nonmedication and medication span annotations.

Multimedia Appendix 4 iCHECK-DH checklist.

## Abbreviations

ADHD: attention-deficit/hyperactivity disorder
AWS: Amazon Web Services
CAMHS: child and adolescent mental health services
CBT: cognitive behavioral therapy
CRIS: Clinical Record Interactive Search
EHR: electronic health record
iCHECK-DH: Guidelines and Checklist for the Reporting on Digital Health Implementations
IE: information extraction
NHS: National Health Service
NLP: natural language processing
NPT: normalization process theory
OHFT: Oxford Health NHS Foundation Trust
VDI: virtual desktop infrastructure
