# Supplementary material for: Integrating Natural Language Processing in Child and Adolescent Mental Health Services Research Using Normalization Process Theory: Implementation Report
Source: JMIR Med Inform. 2026 Oct 26;14:e96370. doi: 10.2196/96370 (PMC13749019; doi:10.2196/96370)
Supplement: Supplementary file 1 [file medinform_v14i1e96370_app1.docx]

**ACECAT-CRIS Annotation Schema and Worked Example**

This is a Multimedia Appendix to a full manuscript published in the J Med Internet Res. For full copyright and citation information see http://dx.doi.org/10.2196/jmir. 96370.

Version 2 (revised following peer review), August 2026

# Scope and general annotation rules

This codebook operationalises the clinical constructs annotated in the ACECAT-CRIS study. It was co-developed by clinicians, researchers, and NLP scientists. An initial draft was piloted on 10 clinical notes and refined through six consensus rounds. The final schema contained 14 primary categories, including Drug, plus three additional medication-attribute labels (Dose, Frequency, and Action), giving 17 span labels in total. Inter-annotator reliability and adjudication procedures are reported in the main manuscript and Multimedia Appendix 4.

1. **Span.** Annotate the shortest contiguous span that preserves meaning. Include status or negation modifiers when required for interpretation.
2. **Polarity.** Affirmed and negated mentions receive the same clinical category. Polarity was not a separate target in the span-model performance reported in the manuscript.
3. **Overlapping labels.** Categories are not mutually exclusive. The same or overlapping text may receive more than one label when the decision rules require it.
4. **Temporality.** Current, past, referred, and waitlisted instances are annotated; relevant status wording is retained within the span.
5. **Attribution.** Problems and support are annotated regardless of whether they are reported by the patient, parent or carer, clinician, school, or another professional.
6. **Pre-annotation.** Pattern-based pre-annotations are annotation aids rather than gold labels; annotators verify, correct, or remove them.
7. **Private care.** Private medication and therapy are recorded separately rather than under Drug or Therapy. Private diagnoses remain within Working_Diagnosis, and privately raised suspicion remains within Suspected_Autism or Suspected_ADHD.
8. **Examples.** Every example in this appendix is a synthetic composite written for illustration. No verbatim patient-record text is reproduced.

**Terminology:** The annotation method is span categorisation rather than document-level multilabel classification. Predictions retain the label and character-level start and end offsets.

# Schema overview and relation to the reported models

The main manuscript reports models for medication names and attributes, five problem categories, psychological therapy, and treatment outcomes. Other schema categories supported cohort identification or were reserved for future model development.

| **Schema label** | **Construct** | **Included in reported span models** |
| --- | --- | --- |
| Drug | Medication name | Yes |
| Dose | Amount per administration | Yes |
| Action | Prescribing action | Yes |
| Frequency | Administration schedule | Yes |
| Therapy | Structured NHS psychological intervention | Yes |
| Other_clin_support | Other NHS clinical support | No |
| Non_clin_support | School, community, social-care, police, or other non-clinical support | No |
| Outcome | Response to treatment or support | Yes |
| Side_effect | Treatment side effect distinct from change in the treatment target | No |
| Problem_Bio | Biological or bodily problem | Yes |
| Problem_Emotional | Emotional problem | Yes |
| Problem_Other | Problem not assigned to biological, emotional, cognitive, or social categories | Yes |
| Problem_Cognitive | Thought, attention, concentration, or cognitive-processing problem | Yes |
| Problem_Socio | Social-functioning problem or adversity in the social environment | Yes |
| Working_Diagnosis | Explicit diagnosis | No |
| Suspected_Autism | Suspected autism or autism assessment pathway | No |
| Suspected_ADHD | Suspected ADHD or ADHD assessment pathway | No |

# Annotation categories

## Drug

**Definition.** The name of any medication prescribed through NHS pathways, whether current or past (for example ‘Phenergan’).

**Medication attributes.** For each Drug span, the following attributes were annotated where documented:

- Dose: the amount given per administration (for example, ‘30 mg’).
- Frequency: the administration schedule (for example, ‘once daily’ or ‘nocte’).
- Action: the clinical action applied to the drug, including initiation, continuation, adjustment, withholding, or discontinuation.

Example: in ‘Started sertraline 25 mg once daily’, annotate ‘sertraline’ as Drug, ‘Started’ as Action, ‘25 mg’ as Dose, and ‘once daily’ as Frequency.

**Decision rules.**

- A generic mention of ‘medication’ alone was not annotated. Qualified forms, such as ‘ADHD medication’ or ‘anti-anxiety medication’, were annotated.
- Drugs that were merely discussed, offered, or under consideration were not annotated; annotation began when prescription or uptake was documented.
- Pattern-based pre-annotations were verified because medication dictionaries could incorrectly match hormones, chemicals, or personal names.
- Where documented, Dose, Frequency, and Action were annotated as separate, potentially overlapping spans.

| **Synthetic note excerpt** | **Annotated span** | **Label(s)** |
| --- | --- | --- |
| Quetiapine 75 mg nocte; GP to offer repeat prescription | Quetiapine | Drug |
| Discontinued Phenergan 25 mg nocte | Phenergan | Drug |
| Fluoxetine capsules 30 mg once daily | Fluoxetine | Drug |

## Therapy

**Definition.** A structured psychological intervention delivered through NHS pathways, whether current, past, referred, waitlisted, or declined. Status modifiers were included when needed to preserve the meaning of the span.

**Decision rules.**

- Care packages such as the Care Programme Approach were annotated as Therapy only when structured psychological work was documented; otherwise they were annotated as Other_clin_support.
- Psychoeducation was annotated as Other_clin_support rather than Therapy.
- Therapy delivered through school arrangements, including through an Education, Health and Care Plan, was annotated as Non_clin_support.
- Private therapy was recorded separately and was not annotated as Therapy.

| **Synthetic note excerpt** | **Annotated span** | **Label(s)** |
| --- | --- | --- |
| He confirms his one-to-one ‘talking therapy’ | one-to-one ‘talking therapy’ | Therapy |
| Family therapy clinic session | Family therapy | Therapy |
| Mum declined further counselling | declined further counselling | Therapy |
| Start CBT work around anger next session | CBT work around anger | Therapy |
| Has previously tried Coping Cat sessions | Coping Cat sessions | Therapy |
| He has had six sessions with his therapist | six sessions with his therapist | Therapy |

## Other_clin_support

**Definition.** NHS clinical support that was neither a drug prescription nor structured psychological therapy, including one-off psychoeducation, parenting groups, dietitian input, sleep-clinic input, crisis planning, GP or emergency-department involvement, and allocation of a CAMHS ‘buddy’ providing reassurance, normalisation, or low-intensity work.

| **Synthetic note excerpt** | **Annotated span** | **Label(s)** |
| --- | --- | --- |
| The plan is to continue attending the parent anxiety group | parent anxiety group | Other_clin_support |
| A crisis plan is in place | crisis plan | Other_clin_support |
| GP saw the patient yesterday | GP | Other_clin_support |

## Non_clin_support

**Definition.** Non-clinical support for the child or young person’s difficulties from school, community, social work, police, or other agencies, including SENCO, SENDIASS, TAS, TAF, and comparable sources of support.

**Decision rules.**

- Therapy delivered through school arrangements, for example through an Education, Health and Care Plan, was annotated here rather than under Therapy.
- Private psychiatry or private therapy was recorded separately and was not annotated here.

| **Synthetic note excerpt** | **Annotated span** | **Label(s)** |
| --- | --- | --- |
| He spoke to someone in student support services | student support services | Non_clin_support |
| He is getting help from the school pastoral service | school pastoral service | Non_clin_support |
| Social care was involved | Social care | Non_clin_support |
| Equine therapy has been added to his EHCP request | Equine therapy; EHCP request | Non_clin_support |

## Outcome

**Definition.** A documented positive, negative, or null response to a treatment or support, including symptom or functional change, engagement, or acceptability. The outcome span was annotated; the treatment to which it referred received its own relevant label.

**Decision rules.**

- A worsening of the treatment target was annotated as a negative Outcome.
- A distinct adverse effect was annotated as Side_effect rather than Outcome.

| **Synthetic note excerpt** | **Annotated span** | **Label(s)** |
| --- | --- | --- |
| Medicine has helped | Medicine has helped | Outcome |
| He is tolerating his medication to beneficial effect | tolerating his medication to beneficial effect | Outcome |
| She said that he felt humiliated after the family therapy session | felt humiliated | Outcome |
| Sleep has improved with promethazine | Sleep has improved | Outcome |

## Side_effect

**Definition.** Any documented side effect of a treatment that was distinct from change in the intended treatment target.

**Decision rules.**

- If the target symptom worsened after treatment, it was annotated as a negative Outcome rather than a Side_effect.

| **Synthetic note excerpt** | **Annotated span** | **Label(s)** |
| --- | --- | --- |
| No side effects were reported | No side effects | Side_effect |
| He replied ‘yawning’, which will be monitored | yawning | Side_effect |
| The medication made her feel tired | feel tired | Side_effect |
| Her appetite is suppressed during the day | appetite is suppressed | Side_effect |

## Problem_Bio

**Definition.** Bodily symptoms or concerns affecting a body part or organ, including tics, startle, shaky hands, heart racing, sensory difficulties, chronic fatigue, chronic pain, allergy or dietary intolerance, neurological abnormality, abnormal hormone or vitamin levels, and body-weight concerns.

**Decision rules.**

- Neurological abnormality was distinguished from neurocognitive or neurodevelopmental difficulty, which was annotated as Problem_Cognitive.
- Tiredness, lethargy, or fatigue was annotated here unless a clear emotional undertone was specified.
- Tics were annotated here even when vocal, owing to their neurological origin.

| **Synthetic note excerpt** | **Annotated span** | **Label(s)** |
| --- | --- | --- |
| The hospital is investigating chronic fatigue | chronic fatigue | Problem_Bio |
| He is not having any physical health issues | not having any physical health issues | Problem_Bio |
| Dyspraxia, motor coordination disorder | Dyspraxia; motor coordination disorder | Problem_Bio; Working_Diagnosis where diagnostic |
| She collapsed in pain during the outburst | collapsed in pain | Problem_Bio |

## Problem_Emotional

**Definition.** Emotional problems, including anxiety, depression, worries, panic, apprehension, fears, and phobias, that affected the child or young person negatively.

**Decision rules.**

- Reactive stress with an identifiable, proportionate stressor was annotated as Problem_Other; generalised stress or stress without an obvious one-off stressor was annotated here.
- Emotional dysregulation was annotated as both Problem_Emotional and Problem_Cognitive.

| **Synthetic note excerpt** | **Annotated span** | **Label(s)** |
| --- | --- | --- |
| This is an anxiety disorder, not just autism-related anxiety | not just autism-related anxiety | Problem_Emotional |
| Objectively, affect was restricted | affect was restricted | Problem_Emotional |
| He is not feeling anxious in social situations | not feeling anxious in social situations | Problem_Emotional |
| He denies feeling low in his mood | denies feeling low | Problem_Emotional |

## Problem_Other

**Definition.** Problems or concerns raised by the patient, clinicians, family, school, or other professionals that were not clearly biological, emotional, cognitive, or social, including conduct or behavioural problems, aggression, outbursts, sleep problems, personality difficulties, and adverse external events such as unspecified trauma or school exclusion.

**Decision rules.**

- Self-harm and suicidal thoughts, intent, or ideation were annotated here so that they remained grouped together.
- Masking or camouflaging behaviours were annotated here.
- Eating-disorder or body-image concerns were annotated here; where cognition or emotion was explicit, the corresponding additional label was applied.
- For obsessive-compulsive phenomena, obsessive thoughts were Problem_Cognitive, compulsive behaviours were Problem_Other, and an unspecified mention of OCD was Problem_Emotional.

| **Synthetic note excerpt** | **Annotated span** | **Label(s)** |
| --- | --- | --- |
| No challenging behaviour in the last six months | No challenging behaviour | Problem_Other |
| Not naughty or violent | Not naughty or violent | Problem_Other |
| He is close to having a fixed-term exclusion | close to having a fixed-term exclusion | Problem_Other |
| Examples of his obsessive-compulsive behaviour | obsessive-compulsive behaviour | Problem_Other |
| No immediate risks to self or others | No immediate risks to self or others | Problem_Other |

## Problem_Cognitive

**Definition.** Thoughts, cognitive processing, attention, or concentration difficulties that adversely affected the child or young person or contributed to mental health difficulties. Hallucinations were also annotated here.

**Decision rules.**

- Attentional or hyperactivity difficulties were annotated here; explicit clinician suspicion of ADHD, referral, or waitlisting was additionally annotated as Suspected_ADHD.
- Emotional dysregulation was annotated as both Problem_Cognitive and Problem_Emotional.

| **Synthetic note excerpt** | **Annotated span** | **Label(s)** |
| --- | --- | --- |
| Preoccupied with negative cognition of self and anxiety-laden thoughts | negative cognition of self; anxiety-laden thoughts | Problem_Cognitive; Problem_Emotional for anxiety-laden thoughts |
| He is struggling to pay attention and concentrate | struggling to pay attention and concentrate | Problem_Cognitive |
| Gory images become more vivid the angrier he gets | gory images | Problem_Cognitive |
| Still experiencing distressing intrusive thoughts | intrusive thoughts | Problem_Cognitive |
| Ongoing severe anxiety and obsessional ruminations | obsessional ruminations | Problem_Cognitive |

## Problem_Socio

**Definition.** Difficulties in social functioning, such as social-communication difficulties, attachment issues, or difficulty starting or maintaining friendships, and adversity in the social environment, such as neglect, bullying, or witnessing domestic violence.

**Decision rules.**

- Social anxiety alone was not a social problem unless a clear functional impact was stated; otherwise it was annotated as Problem_Emotional.

| **Synthetic note excerpt** | **Annotated span** | **Label(s)** |
| --- | --- | --- |
| He shows an insecure attachment | insecure attachment | Problem_Socio |
| Autism-related social interaction and communication difficulties | social interaction and communication difficulties | Problem_Socio |
| Father reported low-level bullying | low-level bullying | Problem_Socio |
| Not interacting with friends | Not interacting with friends | Problem_Socio |

## Working_Diagnosis

**Definition.** An explicit diagnosis, clearly stated as such or expressed as a direct diagnostic term, whether made within CAMHS or privately.

**Decision rules.**

- Clinical impressions entered in diagnosis fields that were not diagnostic terms were annotated under the relevant Problem category rather than Working_Diagnosis.
- Suspicion of a diagnosis or traits alone were not annotated here; Suspected_Autism and Suspected_ADHD were used where applicable.

| **Synthetic note excerpt** | **Annotated span** | **Label(s)** |
| --- | --- | --- |
| Diagnosis: childhood autism; generalised anxiety | childhood autism; generalised anxiety | Working_Diagnosis; Problem_Emotional additionally for contextual anxiety wording |
| Since a private diagnosis of ASD | ASD | Working_Diagnosis |

## Suspected_Autism

**Definition.** Documented clinician suspicion of autism or autistic traits, or referral to or waitlisting for an autism assessment, whether through NHS or private pathways.

**Decision rules.**

- The span was annotated even where suspicion did not result in a diagnosis or an assessment concluded that diagnostic criteria were not met.

| **Synthetic note excerpt** | **Annotated span** | **Label(s)** |
| --- | --- | --- |
| Autism spectrum condition: index of suspicion | Autism spectrum condition: index of suspicion | Suspected_Autism |
| CAMHS plan: ASD assessment | ASD assessment | Suspected_Autism |
| Reassessed for ASD but did not meet diagnostic criteria | reassessed for ASD but did not meet diagnostic criteria | Suspected_Autism |
| Private diagnoses: ADHD, anxiety, possible ASD | possible ASD | Suspected_Autism |

## Suspected_ADHD

**Definition.** Documented clinician suspicion of ADHD or ADHD traits, or referral to or waitlisting for an ADHD assessment, whether through NHS or private pathways.

**Decision rules.**

- The span was annotated even where suspicion did not result in a diagnosis or an assessment concluded that diagnostic criteria were not met.

| **Synthetic note excerpt** | **Annotated span** | **Label(s)** |
| --- | --- | --- |
| ADHD: index of suspicion | ADHD: index of suspicion | Suspected_ADHD |
| CAMHS plan: ADHD assessment | ADHD assessment | Suspected_ADHD |
| He showed evidence of hyperactivity and attentional symptoms | evidence of hyperactivity and attentional symptoms | Suspected_ADHD; Problem_Cognitive for the symptom description |

# Abbreviations

ADHD, attention-deficit/hyperactivity disorder; AP, assistant psychologist; ASD, autism spectrum disorder; CAMHS, Child and Adolescent Mental Health Services; CBT, cognitive behavioural therapy; CPA, Care Programme Approach; CYP, children and young people; DV, domestic violence; EHCP, Education, Health and Care Plan; GP, general practitioner; NHS, National Health Service; OCD, obsessive-compulsive disorder; SENCO, Special Educational Needs Coordinator; SENDIASS, Special Educational Needs and Disabilities Information, Advice and Support Service; TAF, Team Around the Family; TAS, Team Around the School.

# Worked example

The following fictitious composite illustrates the schema end to end. Spans not listed in the table are not annotated.

**Synthetic note:** Seen with mum. He remains on sertraline 50 mg once daily; GP to continue prescription. Ongoing intrusive thoughts and worries; no side effects reported. Sleep has improved since starting medication. Awaiting ASD assessment; school SENCO providing weekly check-ins. Mum declined further family therapy at this time.

| **Annotated span** | **Label** |
| --- | --- |
| sertraline | Drug |
| remains on | Action (continuation) |
| 50 mg | Dose |
| once daily | Frequency |
| GP | Other_clin_support |
| intrusive thoughts | Problem_Cognitive |
| worries | Problem_Emotional |
| no side effects reported | Side_effect |
| Sleep has improved | Outcome |
| Awaiting ASD assessment | Suspected_Autism |
| school SENCO providing weekly check-ins | Non_clin_support |
| declined further family therapy | Therapy |

The generic word ‘medication’ in ‘since starting medication’ is not annotated as Drug because it does not identify a specific medication or a qualified medication class.
