# Supplementary material for: Integrating Natural Language Processing in Child and Adolescent Mental Health Services Research Using Normalization Process Theory: Implementation Report
Source: JMIR Med Inform. 2026 Oct 26;14:e96370. doi: 10.2196/96370 (PMC13749019; doi:10.2196/96370)
Supplement: Supplementary file 2 [file medinform_v14i1e96370_app2.docx]

**Multimedia Appendix: Technical Specification of the NLP Pipeline and Evaluation**

This is a Multimedia Appendix to a full manuscript published in the J Med Internet Res. For full copyright and citation information see http://dx.doi.org/10.2196/jmir. 96370.

# System boundary and data flow

1. Pseudonymised free-text clinical notes were accessed through the Clinical Record Interactive Search system on the Akrivia Health platform within Oxford Health NHS Foundation Trust's Trusted Research Environment. Annotation, model training, inference, and analysis were conducted within this secure environment.
2. The target concepts were manually annotated as labelled text spans with character-level start and end offsets. The reference annotations used for supervised model development and held-out evaluation were consensus-adjudicated.
3. The standard pretrained RoBERTa-base checkpoint was obtained through the Hugging Face Transformers framework [1,2], stored locally within the Trusted Research Environment, and loaded through the spacy-transformers component. The RoBERTa encoder and spaCy SpanCategorizer were fine-tuned jointly using the consensus-adjudicated model-development annotations. No additional masked-language-model pretraining or other domain-adaptive pretraining was performed on CRIS clinical notes.
4. Before model processing, long notes were divided into nonoverlapping chunks to support note-level offset management and processing within the secure environment. Notes were first divided at paragraph boundaries. A paragraph boundary was defined as a blank line represented by at least two consecutive line-break sequences, including '\n\n', '\r\n\r\n', or the legacy form '\r\r'. If a resulting paragraph segment still exceeded the tokenised sequence length accepted by RoBERTa, it was divided into consecutive nonoverlapping segments until each segment was within the checkpoint's maximum sequence length of 512 subword tokens. These external paragraph-based chunks were processed independently.
5. Within each external chunk, the spacy-transformers component generated contextual representations using overlapping windows of 128 spaCy tokens with a stride of 96 spaCy tokens. The window and stride values refer to spans of spaCy tokens supplied to the transformer component; they are not embedding dimensions and are distinct from RoBERTa's 512-subword-token architectural limit. Because the stride was smaller than the window, adjacent internal windows overlapped to provide additional left and right context [3].
6. Predicted chunk-level spans were converted to character offsets in the corresponding source note and stored with the predicted label, source-note identifier, and associated metadata. The external chunks were nonoverlapping; therefore, no cross-chunk voting or duplicate-resolution procedure was required. No document-level classification or note-level label aggregation was performed.
7. After model selection, the independent patient-disjoint test set was used once for final evaluation. A predicted span was considered correct only when its label and both character boundaries exactly matched a consensus-adjudicated reference span. No partial credit was assigned for overlapping spans or approximately matching boundaries.

# Cohort identification and sampling

The OHFT CRIS team identified records dated 2011-2022. Eligibility could be established from structured diagnostic codes or anxiety-related information in free text. The structured-code definitions supplied for this revision are listed below.

As stated in the main text, to maximise sensitivity during initial record retrieval, the OHFT CRIS team first created a broad candidate cohort using a union criterion that contained evidence of autism or ADHD, evidence of anxiety problems, or both: (1) evidence of autism, as recorded by the following ICD-10 codes; or ADHD, as recorded by the following ICD-10 codes, specified in the table below; (2) evidence of anxiety problems as recorded either as formal anxiety diagnoses by ICD-10 codes, or mentions of anxiety-related keywords in clinical notes, as specified in the table below. In line with OHFT CAMHS service thresholds, records of eligible children aged 6-18 who accessed services in OHFT were retrieved.

Structured identifiers:

| **Route** | **Operational definition** | **Status** |
| --- | --- | --- |
| Autism | ICD-10 F84.0, F84.1, or F84.5 | Specified |
| ADHD/hyperkinetic disorders | ICD-10 F90.0-F90.1, F90.8, or F90.9 | Specified |
| Anxiety disorders | ICD-10 F40.x-F41.x | Specified |

# Free-text search strings:

| **Route** | **Operational definition** | **Status** |
| --- | --- | --- |
| Anxiety disorders | Anxious, anxiety, apprehend*, fear*, nervous*, stress*, phobi* | Specified |

# Annotated corpus and partitioning

Four clinical researchers annotated 970 notes. Ten notes used for initial schema piloting and refinement were excluded from quantitative analysis. The remaining 960 notes from 419 patients were assigned either to model development and evaluation or to the separate inter-annotator reliability analysis.

The model corpus comprised 697 notes from 302 patients. Before model development, patients were divided into a 549-note model-development partition and a 148-note held-out test partition. No patient contributed notes to both partitions. The 263 notes used for the pre-adjudication inter-annotator reliability analysis were separate from the model corpus and were not used for supervised model training or held-out testing.

| **Purpose** | **Notes** | **Patients** | **Number of annotators** | **Use** |
| --- | --- | --- | --- | --- |
| Schema development | 10 | 7 | 2 | Piloting and schema refinement; excluded from quantitative analysis |
| Model development and internal selection | 549 | 226 | 2 | training spans, subsequently divided into 440 training notes and 109 internal development notes |
| Held-out test | 148 | 76 | 2 | Patient-disjoint final evaluation; used once |
| Inter-annotator agreement (IAA) analysis | 263 | 117 | 4 | Pre-adjudication annotations; separate from model corpus |
| Total after schema-development exclusion | 960 | 419 | / | 697 model notes plus 263 IAA notes |

The 697-note model corpus, comprising the model-development and held-out test partitions, contained 494,963 words. Median note length was 527 words (IQR 261–925). These word counts are descriptive and are not equivalent to RoBERTa subword-token counts.

# Target labels

| **Domain** | **Model label** | **Number of labels** |
| --- | --- | --- |
| Medication | Drug, Dose, Action, Frequency | 4 |
| Problems | Problem_Cognitive, Problem_Emotional, Problem_Socio, Problem_Bio, Problem_Other | 5 |
| Psychological treatment | Therapy | 1 |
| Treatment response | Outcome | 1 |
| **Total** |  | 11 |

# The Outcome label captured documented positive, negative, or null responses to treatment or support, including symptom or functional change, nonresponse, engagement, and acceptability. Side effects were annotated separately using the Side_effect schema label and were not included among the 11 labels evaluated in this report. Problem_Other was treated as one aggregated problem label. The SpanCategorizer permitted overlapping spans and allowed the same candidate span to receive more than one label when multiple label scores met the decision threshold.

# The full annotation schema comprised 14 clinical categories and three additional medication attributes, giving 17 span labels in total. The following six schema labels were not target labels in the reported model evaluation: Side_effect, Other_clin_support, Non_clin_support, Working_Diagnosis, Suspected_Autism, and Suspected_ADHD. These six labels were retained in the annotation schema for descriptive annotation or future model development but were not included in the model evaluation reported here.

# Model architecture and long-note handling

The candidate-span generator proposed spans containing between 1 and 20 spaCy tokens. Consequently, a reference span longer than 20 spaCy tokens was outside the candidate space and could not be recovered as a single predicted span. Similarly, because external chunks were processed independently, a span crossing an external chunk boundary could not be predicted as one continuous span.

| **Component** | **Specification** |
| --- | --- |
| Task | Supervised categorisation of potentially overlapping text spans |
| Pipeline | spacy-transformers transformer component followed by spaCy SpanCategorizer |
| Base encoder | Standard pretrained RoBERTa-base checkpoint |
| RoBERTa architecture | 12 transformer layers, 768 hidden units, and 12 attention heads |
| Architectural maximum | 512 RoBERTa subword tokens per encoded sequence |
| Supervised targets | Consensus-adjudicated labelled spans from the model-development partition |
| Candidate-span generator | All contiguous spans containing 1-20 spaCy tokens, inclusive |
| Span representation | Mean-maximum reducer with a hidden-layer size of 128 |
| Span classification | Independent logistic outputs for the target labels |
| Decision threshold | Global probability threshold of 0.5 |
| Maximum positive labels | Unrestricted (max_positive=null) |
| Long-note preprocessing | Nonoverlapping external chunks created at paragraph boundaries and subdivided where required |
| Internal transformer processing | Overlapping windows of 128 spaCy tokens with a stride of 96 spaCy tokens |
| Output | Predicted label, note-level character offsets, source-note identifier, and associated metadata |

# Software environment

Annotation and data export used Prodigy version 1.11.14. Model training, inference, and evaluation used Python 3.10 and the following principal packages:

| **Software** | **Version** |
| --- | --- |
| Python | 3.10 |
| spaCy | 3.7.6 |
| spacy-transformers | 1.3.5 |
| PyTorch | 2.3.1 |
| Hugging Face Transformers | 4.36.2 |
| NumPy | 1.26.4 |
| pandas | 2.2.2 |
| scikit-learn | 1.5.1 |
| seaborn | 0.13.2 |
| Prodigy | 1.11.14 |

Prodigy annotation/export and spaCy model training were separate stages of the workflow. The reported versions describe the retained software environments used for these respective stages.

**Supervised optimisation**

| **Parameter** | **Configuration** |
| --- | --- |
| Optimiser | Adam |
| β1 | 0.9 |
| β2 | 0.999 |
| ε | 1 × 10⁻⁸ |
| L2 weight decay | 0.01 |
| Gradient clipping | 1.0 |
| Learning-rate schedule | Linear warm-up followed by linear decay |
| Peak learning rate | 5 × 10⁻⁵ |
| Warm-up | 250 optimiser-update steps |
| Total learning-rate schedule | 20,000 optimiser-update steps |
| Dropout | 0.1 |
| Gradient accumulation | Three batches per optimiser update |
| Maximum training steps | 20,000 |
| Epoch-based limit | None (max_epochs=0) |
| Random seed | 0 |
| Frozen components | None |
| Mixed-precision training | Disabled |
| Transformer maximum padded batch items | 4,096 |
| Training batcher | Padded batcher with size parameter 500, buffer 256, and oversized-batch discarding enabled |
| Loss | Default spaCy SpanCategorizer loss; no custom loss or class weighting |

The learning rate increased during the first 250 optimiser-update steps to the configured peak of 5 × 10⁻⁵ and then decayed linearly according to the 20,000-step schedule. The 20,000-step value was the maximum training limit and learning-rate schedule length, not necessarily the update step at which the selected checkpoint was obtained.

**Validation and model selection**

The model-development partition comprised 549 notes from 226 patients. When these annotations were exported for training, Prodigy's eval_split=0.20 setting randomly reserved 20% of the notes for internal development and model selection and allocated the remaining 80% to supervised training. This internal split was performed at the note level. Consequently, different notes belonging to the same patient could occur in the internal training and development subsets.

The exported datasets contained 440 training notes and 109 development notes. Development-set performance was evaluated every 200 optimiser-update steps. Early stopping used a patience of 1,600 update steps without improvement, corresponding to eight evaluation intervals.

spaCy retained the checkpoint with the highest overall development-set score. Under the retained configuration, this score was determined solely by development-set span F1 because the score weight was 1.0 for span F1 and 0.0 for span precision and recall. The global probability threshold was fixed at 0.5 in the configuration and was not tuned.

The independent held-out test set comprised 148 notes from 76 patients. It was patient-disjoint from the complete 549-note model-development partition and was not used for early stopping, threshold setting, hyperparameter selection, or checkpoint selection. It was used only for final evaluation after the model and decision rule had been selected.

**Decision threshold and prediction storage**

A single global probability threshold of 0.5 was applied to all labels. No label-specific thresholds were used. Because max_positive was set to null, more than one label could be assigned to the same candidate span when the respective scores met the threshold. Overlapping candidate spans could also be retained.

Predicted spans meeting the threshold were stored under the spaCy span key sc, together with their labels and scores. Chunk-relative offsets were subsequently converted to character offsets in the original source note.

# Model evaluation

A predicted span was counted as a true positive only when its label and start and end character offsets exactly matched a consensus-adjudicated reference span. Unmatched predictions were false positives and unmatched reference spans were false negatives.

| **Metric** | **Calculation** | **Interpretation** |
| --- | --- | --- |
| Precision | TP / (TP + FP) | Proportion of predicted spans that exactly matched a reference span |
| Recall | TP / (TP + FN) | Proportion of reference spans recovered by an exact prediction |
| F1 | 2 x precision x recall / (precision + recall) | Harmonic mean of precision and recall |
| Macro average | Unweighted mean across labels | Each label contributes equally, regardless of its number of reference spans |

Label-specific and macro-average metrics were recomputed in 5,000 patient-level cluster-bootstrap replicates. Patients, rather than individual notes or spans, were resampled with replacement, and all notes belonging to a sampled patient were retained. The reported 95% percentile confidence intervals were the 2.5th and 97.5th percentiles of the bootstrap estimates. The held-out test set was used once for final evaluation and was not used for model selection or optimisation.

# Inference environment and throughput

| **Item** | **Details** |
| --- | --- |
| Environment | Akrivia/CRIS Trusted Research Environment hosted in an AWS virtual desktop infrastructure |
| Reported CPU | Intel Xeon Platinum 8488C, 2.4 GHz |
| Reported memory | 32 GB RAM |
| Observed model throughput | Approximately 3 notes per second |
| Human annotation comparator | Approximately 4 minutes per note |
| Interpretation | Approximately 700-fold higher throughput; this is an operational comparison rather than a controlled benchmark |

# Scope and limitations of the technical evaluation

The evaluation concerns secondary research extraction within one local corpus; it is not evidence of suitability for clinical decision-making. The held-out test set was patient-disjoint from the complete model-development partition, but this does not constitute external validation. The internal split of the 549-note model-development partition into 440 training notes and 109 development notes was performed at the note level, meaning that different notes from the same patient could occur in both internal subsets. This may have influenced checkpoint selection, although the independent test set remained patient-disjoint from the entire model-development partition. The model was trained once using a single random seed. The patient-level bootstrap confidence intervals therefore quantify uncertainty arising from resampling patients in the held-out test set but do not quantify variability attributable to random model initialisation or repeated training. Strict matching penalises clinically plausible predictions when their character boundaries differ from the adjudicated reference boundaries. Performance varies substantially between labels; macro averages should not replace label-specific results. Polarity, temporality, treatment-to-outcome relation extraction, and coreference were not separate targets in the reported span-performance tables unless explicitly added and evaluated elsewhere. A span crossing a paragraph-based chunk boundary could not be predicted as one continuous span.

**References**

**1.** Honnibal M, Montani I, Van Landeghem S, Boyd A. spaCy: Industrial-strength Natural Language Processing in Python. 2020. doi:10.5281/zenodo.1212303.

**2.** Wolf T, Debut L, Sanh V, et al. Transformers: State-of-the-Art Natural Language Processing. Proceedings of EMNLP: System Demonstrations. 2020:38-45. doi:10.18653/v1/2020.emnlp-demos.6.

**3.** Liu Y, Ott M, Goyal N, et al. RoBERTa: A Robustly Optimized BERT Pretraining Approach. arXiv. 2019;arXiv:1907.11692.
