# Supplementary material for: Integrating Natural Language Processing in Child and Adolescent Mental Health Services Research Using Normalization Process Theory: Implementation Report
Source: JMIR Med Inform. 2026 Oct 26;14:e96370. doi: 10.2196/96370 (PMC13749019; doi:10.2196/96370)
Supplement: Supplementary file 3 [file medinform_v14i1e96370_app3.docx]

**Multimedia Appendix. Category-specific Krippendorff's alpha for nonmedication and medication span annotations**

This is a Multimedia Appendix to a full manuscript published in the J Med Internet Res. For full copyright and citation information see http://dx.doi.org/10.2196/jmir. 96370.

Notes: Inter-annotator reliability was calculated from pre-adjudication annotations of 263 notes using position-based nominal Krippendorff's alpha. The text was split at every observed span boundary into nonoverlapping character intervals. For each label, each interval was coded by each of the 4 annotators as covered or not covered by a span of that label. Analysis was restricted to text covered by at least one included annotation. Intervals were weighted by character length, so disagreements in span boundaries were penalised in proportion to the nonoverlapping text. This method assesses reliability of labelled character coverage and is not Krippendorff's unitising alpha. Ninety-five percent percentile confidence intervals were estimated using 5,000 patient-level cluster-bootstrap replicates, retaining all notes belonging to each sampled patient. All 5,000 replicates produced valid category-specific estimates.

| **Labels** | | **Estimate** | **95% CI** |
| --- | --- | --- | --- |
| **Medication Treatment** | drug names | 0.967 | 0.955-0.979 |
|  | dose | 0.926 | 0.892-0.959 |
|  | action | 0.665 | 0.579-0.743 |
|  | frequency | 0.927 | 0.881-0.965 |
| **Problems** | cognitive | 0.325 | 0.096-0.418 |
|  | emotional | 0.579 | 0.477-0.635 |
|  | social | 0.516 | 0.280-0.688 |
|  | biological | 0.411 | 0.318-0.526 |
|  | other | 0.418 | 0.271-0.467 |
| **Psychological Therapy** |  | 0.546 | 0.409-0.699 |
| **Outcomes** |  | 0.343 | −0.028-0.549 |
