# Supplementary material for: Integrating Natural Language Processing in Child and Adolescent Mental Health Services Research Using Normalization Process Theory: Implementation Report
Source: JMIR Med Inform. 2026 Oct 26;14:e96370. doi: 10.2196/96370 (PMC13749019; doi:10.2196/96370)
Supplement: Supplementary file 4 [file medinform_v14i1e96370_app4.docx]

**Completed iCHECK-DH Reporting Checklist**

This is a Multimedia Appendix to a full manuscript published in the J Med Internet Res. For full copyright and citation information see http://dx.doi.org/10.2196/jmir. 96370.

Version 3 (revised following editorial), September 2026

# Checklist

| **Item** | **Requirement** | **Status** | **Reported location** | **How addressed** |
| --- | --- | --- | --- | --- |
| 1 | Title (M) | Reported | Title | Identifies the paper as an implementation report and names the NLP intervention, setting, and NPT framework. |
| 2 | Abstract (M) | Reported | Abstract | Structured abstract reports the intervention, seven-phase strategy, implementation framework, coverage, annotation reliability, model performance, implementation findings, and intended use. |
| 3 | Context (M) | Reported | Introduction: Context; Methods: Target Population and Setting | Describes the NHS CAMHS setting, target population, organisational context, and implementation stage. |
| 4 | Problem statement (M) | Reported | Introduction: Problem Statement | Explains limited use of routine clinical text, under-recording in structured fields, and service-level constraints motivating the implementation. |
| 5 | Similar interventions (M) | Reported | Introduction: Similar Interventions | Positions the work within established EHR text-mining research and identifies the added contribution as implementation learning within a secure CAMHS research setting. |
| 6 | Aim and objectives (M) | Reported | Abstract: Objectives; Methods: Research Questions | States the implementation aim, clinical research questions, and quantitative and qualitative evaluation outcomes. |
| 7 | Blueprint summary (M) | Reported | Methods: Blueprint Summary; Figure 1 | Describes the seven implementation phases, key adaptations, and the NPT-informed roadmap. |
| 8 | Technical design (M) | Reported | Methods: Technical Architecture and Key Features; NLP Model Development and Evaluation; Multimedia Appendix 3 | Describes the secure architecture, software, span-categorisation approach, model encoder, long-note handling, output structure, and research-only status. |
| 9 | Target (M) | Reported | Methods: Target Population and Setting | Defines the implementation users, service setting, research population, eligibility criteria, and intended beneficiaries. |
| 10 | Data (M) | Reported | Methods: Data Governance and Privacy; Ethical and Regulatory Considerations | Describes pseudonymisation, secure access, storage, processing, governance oversight, and restrictions on data removal. |
| 11 | Interoperability (M) | Reported | Methods: Interoperability with Clinical Systems | Describes aggregation from CareNotes and RiO through CRIS/Akrivia and adaptation of the pipeline to the CRIS data schema. |
| 12 | Participating entities (M) | Reported | Methods: Technical Architecture; Target Population and Setting; Methods: Availability and intended use; Data Governance and Privacy; Acknowledgments | The implementing organisation, data platform, collaborators, and funders are identified. OHFT owns the dataset that underlined the development and validation of the pipeline. The Intellectual property of the pipeline is owned by the University of Oxford and managed operationally by OHFT’s CRIS team. |
| 13 | Budget planning (M) | Reported | Results: Implementation Process Outcomes; Lessons Learned | Resource constraints, annotation workload, computing limitations, and delays are described in Implementation and lessons learnt sections. The budget associated with maintaining the CRIS dataset the TRE where the server used resided within was covered by the OHFT CRIS team, and were free for approved research use. |
| 14 | Sustainability (M) | Partly reported | Methods: Availability and Intended Use; Results: Lessons Learned; Discussion | Code availability within the TRE, infrastructure needs, and requirements for wider deployment are discussed. Reusability of this pipeline on other projects were not formally assessed due to the time constraint associated with this project. |
| 15 | Coverage (M) | Reported | Methods: Target Population and Setting; Results: Outcomes of the Implementation | Reports the OHFT geographical coverage and the numbers of patients and clinical notes processed. |
| 16 | Outcomes (M) | Reported | Abstract: Results; Results: Outcomes of the Implementation; Technical Evaluation Outcomes | Reports implementation process outcomes, annotation reliability, model performance, throughput, and implementation barriers. |
| 17 | Lessons learned (M) | Reported | Results: Lessons Learned; Tables 4-5 | Reports facilitators, barriers, adaptations, resource implications, and recommendations for future implementations. |
| 18 | Unintended consequences (NM) | Reported | Abstract: Results; Results: Implementation Process Outcomes | Reports no major adverse events or harms and identifies unplanned delays, reconfiguration work, and reduced engagement during prolonged annotation. |
| 19 | Discussion (M) | Reported | Discussion | Summarises the main findings, interpretation, limitations, generalisability, intended use, and future implications. |
| 20 | General (NM) | Reported | Methods: Ethical and Regulatory Considerations; Acknowledgments; Methods: Availability and intended use; Conflicts of Interest, Funding and Author contribution declaration sections | Code availability, governance and ethical review arrangements and the published protocol are described. The manuscript also included a complete conflicts-of-interest statement and any journal-required funding and data/code availability declarations. |

# Source

Perrin Franck C, Babington-Ashaye A, Dietrich D, et al. iCHECK-DH: Guidelines and Checklist for the Reporting on Digital Health Implementations. J Med Internet Res. 2023;25:e46694. doi:10.2196/46694.
